# International Overdose Awareness Day — August 31, 2019

**DOI:** 10.15585/mmwr.mm6834a1

**Published:** 2019-08-30

August 31, 2019, is International Overdose Awareness Day, a global event that aims to raise awareness that overdose death is preventable and to reduce the stigma associated with drug-related death. Goals also include providing information about risk for overdose and community services and preventing drug-related harm through evidence-based policy and practice (https://www.overdoseday.com).

The opioid overdose epidemic, which killed 47,600 U.S. persons in 2017,* substantially expanded in 2013 driven by rapid increases in overdose deaths involving synthetic opioids (excluding methadone), particularly illicitly manufactured fentanyl.^†^ Cocaine and methamphetamine overdose deaths co-involving synthetic opioids also rapidly increased during this period (*1*).

A report in this issue of *MMWR* documented decreases in opioid-involved overdose deaths in 25 states from July–December 2017 to January–June 2018, especially those involving fentanyl analogs and prescription opioids. Overdose deaths involving illicitly manufactured fentanyl (including those co-occurring with illicit opioids and stimulants) increased (*2*). Improved identification of persons at high risk for overdoses involving illicitly manufactured fentanyl and linkage to risk-reduction services and evidence-based treatment are critical to reducing opioid deaths. Further information on CDC’s state efforts and overdose data is available at https://www.cdc.gov/drugoverdose/index.html.

## Footnotes

* https://www.cdc.gov/drugoverdose/data/statedeaths.html.

† https://www.cdc.gov/drugoverdose/epidemic/index.html.
